# Martial arts increase oxytocin production

**DOI:** 10.1038/s41598-019-49620-0

**Published:** 2019-09-10

**Authors:** Yuri Rassovsky, Anna Harwood, Orna Zagoory-Sharon, Ruth Feldman

**Affiliations:** 1 Department of Psychology and Gonda Multidisciplinary Brain Research Center, Bar-Ilan University, Ramat-Gan, Israel; 2 Department of Psychiatry and Biobehavioral Sciences, UCLA Semel Institute for Neuroscience and Human Behavior, Los Angeles, CA USA; 3 Center for Developmental, Social, and Relationship Neuroscience, Interdisciplinary Center, Herzliya, Israel

**Keywords:** Human behaviour, Cooperation

## Abstract

Numerous studies have demonstrated that oxytocin (OT), a peptide hormone, plays an important role in regulating mammalian social behaviors, linking it to social affiliation in parent-infant attachment, romantic and filial relationships, and other prosocial behaviors, such as trust and cooperation. Not surprisingly, research efforts have been made to increase endogenous levels of OT. In the present study, we investigated whether traditional martial arts training, which integrates the natural benefits of physical exercise with dyadic prosocial interaction, would result in OT response. To this end, 68 beginner and advanced participants were recruited from several schools practicing Jujitsu (“soft art”), a form of traditional martial arts originating in Japan. Salivary OT levels were assessed at baseline, immediately following high-intensity training, and following a cool-down period. Analyses revealed a significant increase in OT immediately after a high-intensity training, returning to baseline levels following a cool-down period. Additionally, although no significant difference between beginner and advanced martial artists was found, a significantly higher increase in salivary OT followed ground grappling, as compared to “punch-kick” sparring, indicating an added benefit of close contact tactile interaction. These results suggest that the reportedly socially beneficial effects of traditional martial arts may be in part mediated by OT release and underscore the potentially therapeutic applications of these methods for disorders involving social dysfunction, such as autism, conduct problems, or schizophrenia.

## Introduction

Oxytocin (OT) is a peptide hormone that plays an important role in regulating mammalian social behaviors^1^. In animals, OT has been shown to support the formation of attachment bonds^2^. Studies have shown, for example, that OT mediated maternal behaviors, such as licking and grooming in rats^3^, olfactory recognition of offspring in sheep^4^, and the grooming and contact of Rhesus Macaques^5^. These effects are paralleled in humans, linking OT to social affiliation in parent-child attachment^6,7^. For instance, synchronous interactions that involve physical touch between parents and young children were shown to increase endogenous OT production in both healthy infants^6^ and preschoolers with autism spectrum disorders^8^. Subsequent studies also showed OT release in romantic and filial relationships^9,10^, as well as other prosocial behaviors, such as trust and cooperation^11^.

These potentially beneficial effects of OT have naturally resulted in efforts to increase endogenous levels of OT. Although several studies have reported therapeutic effects of oral, intravenous, and intranasal administration of OT in disorders of social dysfunction, such as autism and schizophrenia^12–14^, substantial challenges remain regarding the passage of OT through the blood-brain barrier^15^. As such, there is a continued need to explore non-pharmacological approaches for increasing endogenous OT. A potentially promissing way to naturally increase OT levels is through physical exercise. Indeed, several studies have suggested that exercise-induced increases in OT may be important for modulating cardiovascular changes and fluid homeostasis during and following exercise and may also moderate stress-induced response. For example, an early study in rats has shown that OT is released in the complex involving the nucleus of the solitary tract and the dorsal motor nucleus of the vagus to restrain exercise-induced tachycardia^16^. A more recent study reported that forced swimming in rats induced OT release into the blood plasma and the hypothalamic paraventricular nucleus^17^. A few studies in humans suggested similar results. Two small trials in healthy participants reported an increase in OT following a prolonged running exercise^18,19^, and a recent study showed salivary OT concentrations increases following moderate 10-minute running, remaining significantly above baseline 40 minutes after completion of the exercise^20^.

The beneficial effects of physical exercise on physical, cognitive, and emotional well-being are well documented in healthy individuals^21^, as well as many medical and psychiatric illnesses^22–24^. This ever-increasing perception of physical exercise as medicine is nicely illustrated in the citation from an interview with Dr. Robert Sallis, the president of the American College of Sports Medicine, stating that “if we had a pill that conferred all the proven health benefits of exercise, physicians would widely prescribe it to their patients and our healthcare system would see to it that every patient had access to this wonder drug”^25^. One type of sport that confers the benefit of physical exercise and involves dyadic prosocial interaction is traditional martial arts. Over the past half century, martial arts have gained increasing popularity in the West, as their positive effects on cognitive functions, self-regulation, and sense of well-being have been demonstrated^26,27^. The philosophy underpinning traditional martial arts is one of attaining the Zen state of mushin (“no mindedness”). This describes a state whereby the participant is capable of “fighting” to their fullest extent but without aggressive feelings. Such balance is achieved through ritualization of combat moves and the requirement of respect to the instructor, practice space, and one another, as well as by highlighting the importance of meditation and philosophies such as peace, benevolence, humanity, and self-restraint^28^.

Research into the martial arts has focused on those elements that are most valuable to the targeted population. Research with adolescents and young adults look at the benefits of martial arts in teaching self-control, enhancing self-esteem, teaching a more positive response to physical challenges, and inducing greater emotional stability, self-confidence, and assertiveness. Martial arts provide an outlet for participants to channel energy into a productive and self-enhancing activity^29^. They have also been demonstrated to improve concentration and self-awareness in children^30^ and enhance executive functions^31^, including self-monitoring, awareness^32^ and cognitive-regulation^30^.

In the present study, we examined the effects of martial arts training on OT response. To this end, beginner and advanced participants were recruited from several schools practicing Jujitsu (“soft art”): Dennis Survival Jujitsu (DSJJ) and Brazilian Jujitsu (BJJ). Both approaches have originated from Japanese martial art and integrate the aforementioned aspects of traditional martial arts into their practice. Additionally, both forms include a *randori* component (high-intensity, free-style friendly tournament) in each class. However, whereas *randori* in DSJJ typically involves “punch-kick” sparring, BJJ focuses on ground grappling. Thus, we sought to address the following three questions. First, given the early suggestions connecting physical exercise and OT, we examined whether the high-intensity aerobic training during martial arts would result in exercise-induced increases in OT. Second, as beginner and advanced participants have had substantially different levels of prior martial arts training, we investigated whether this might lead to differential OT responses. Finally, we examined whether the longer close contact time occurring during ground grappling would result in greater OT response.

## Results

**Figure 1:**
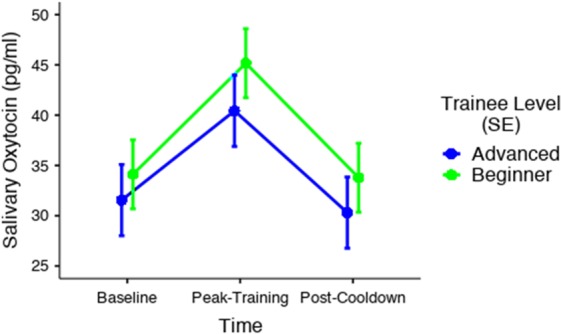
Comparison of salivary oxytocin response between beginner and advanced martial artists during martial arts training. A significant change from baseline to peak-training, as well as from peak-training to post-cooldown, was found for both groups. However, neither the main effect of belt level, nor the interaction between time of saliva collection and belt level, were statistically significant.

To examine the OT response between beginner and advanced martial artists, LMM was conducted, with trainee level (beginner vs. advanced), time of saliva collection (baseline, peak-training, cool-down), and their interactions as fixed factors, and an intercept for subject as a random factor. These analyses demonstrated a significant effect of time of saliva collection, *F* (2, 120) = 12.0, *p* < 0.001, with post-hoc comparisons indicating a significant change from baseline to peak-training (*p*_bonferroni_ < 0.001), as well as from peak-training to post-cooldown, (*p*_bonferroni_ < 0.001). However, neither the main effect of belt level, *F* (1, 60) = 0.79, *p* = 0.38, nor the interaction between time of saliva collection and belt level, *F* (2, 120) = 0.10, *p* = 0.91, were statistically significant (see Fig. 1).

**Figure 2:**
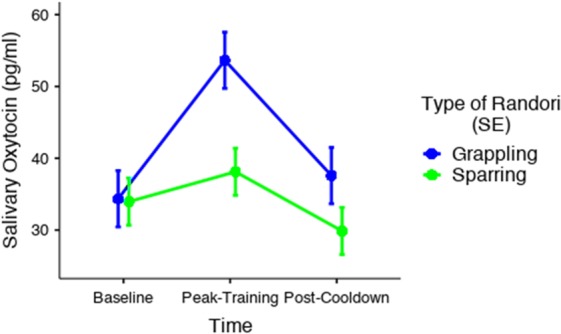
Comparison of salivary oxytocin response between grappling and sparring sessions of martial arts. A significant change from baseline to peak-training, as well as from peak-training to post-cooldown, was found for both groups. Whereas the main effect of type of *randori* did not reach statistical significance, the interaction between time of saliva collection and type of randori was significant, reflecting a significant difference in OT response between grappling and sparring immediately following peak-training.

LMM was then employed to examine potential differences in OT response between the different types of training across time, such that type of *randori* (sparring vs. grappling), time of saliva collection (baseline, peak-training, cool-down), and their interactions were entered as fixed factors and an intercept for subject as a random factor. These analyses revealed a significant effect of time of saliva collection, *F* (2, 132) = 15.6, *p* < 0.001, with post-hoc comparisons again indicating a significant change from baseline to peak-training (*p*_bonferroni_ < 0.001), as well as from peak-training to post-cooldown, (*p*_bonferroni_ < 0.001). Furthermore, whereas the main effect of type of *randori* did not reach statistical significance, *F* (1, 66) = 3.47, *p* = 0.07, the interaction between time of saliva collection and type of *randori* was significant, *F* (2, 132) = 4.69, *p* = 0.01, (see Fig. 2). As can be seen in Fig. 2, further exploration of the interaction through post-hoc comparisons of the simple main effects indicated that a significant difference in OT response between grappling (*M* = 53.6, *SE* = 6.28) and sparring (*M* = 38.1, *SE* = 2.39) occurred only immediately following peak-training (*p*_bonferroni_ = 0.04). This difference was further confirmed by comparing the areas under the curve, such that the AUC_G_ in the grappling condition (*M* = 2409, *SE* = 677) was significantly larger than the AUC_G_ in the sparring condition (*M* = 731, *SE* = 147.5, *p* = 0.006). Similarly, the AUC_I_ in the grappling condition (*M* = 940, *SE* = 371) was significantly larger than the AUC_I_ in the sparring condition (*M* = 124, *SE* = 75.6, *p* = 0.01).

## Discussion

The current study was a pioneering effort to examine whether a session of traditional martial arts training would induce an increase in OT levels. We found a significant increase in OT immediately after a high-intensity *randori* session, returning to baseline levels following a cool-down period. Additionally, although no significant difference between beginner and advanced martial artists was found, a significant interaction between time of saliva collection and type of *randori* indicated that grappling resulted in a significantly higher OT increase than sparring. Given the beneficial effects of traditional martial arts on cognitive and psychological functions^30,31^, and the reported therapeutic effects of exogenous administration of OT in disorders of social dysfunction, such as autism and schizophrenia^12–14^, this readily available, non-invasive training program may have wide implications for physical fitness and community health.

Several potential mechanisms may underlie OT release during *randori*. As mentioned above, a few animal and human studies have reported OT increase following aerobic exercise^17,20^. Thus, it is possible that the high-intensity physical activity occurring during *randori* may, in itself, lead to increases in OT. However, another more complex social mechanism that may contribute to OT increase during *randori* is the ability of OT to increase the salience of social information^33^. Early studies in sheep, for example, reported that OT promotes the selective olfactory recognition of offspring^4^. In humans, administered OT was found to increase gaze toward eye regions, which are considered the most socially communicative part of faces^34^. Moreover, several neuroimaging studies have shown that the administration of OT can have effects on socially-relevant brain areas including the amygdala^35^ and the ventral tegmental area^33^, and studies using fMRI^36^ found that the tendency toward increasing gaze to the eyes is associated with an increased coupling of amygdala and superior colliculi activity, supporting the view that OT biases an individual toward social visual information.

These key features of increased social information salience are crucial in the interpersonal synchrony, which has been associated with OT function. The OT molecule was shown to be critically involved with repetitive synchronous behaviors in a range of living organisms, from coordination of reproductive behavior in roundworms to flocking behavior in birds^37^. Furthermore, in humans, it has been demonstrated that increased levels of OT are related to increased synchronous gaze between mothers and their children^38^ and neural synchrony during social coordination^39^. Thus, the emphasis on dyadic synchronous behaviors in martial arts may contribute to increases in salivary OT. Of course, additional studies are needed to examine whether this synchronous behavior common in martial arts results in greater OT release, as compared to other, solitary physical activities described above^20^.

It should be noted that although the presumably OT-mediated bias toward social information has commonly been associated with prosocial behaviors, several studies have reported OT-related antisocial behaviors, especially in the context of competitive situations^40^. For instance, increases in OT have been associated with increased parochial altruism, or the drive towards “tend and defend”^41^. This refers to the promotion of prosocial behaviors and empathetic responses to the in-group, while increasing defensive actions, although not proactive aggression, towards the out-group. This phenomenon has been demonstrated in hypothetically threatening^41^ and unpredictably startling^42^ laboratory situations. Additionally, antisocial behaviors following increased OT have been observed in threatening situations, such as protecting the young, mating, and out-group conflict^40^. Interestingly, in a recent study comparing a combat-trained and control non-combat male veterans, OT administration was found to attenuate the response to combat stimuli and increased responses to pleasant social scenes, whereas for non-combat controls OT administration increased social salience when presented with combat-related cues^43^. Thus, it appears that both prosocial and antisocial, OT-mediated, responses may be strongly related to contextual and individually predisposing factors, making their universal applicability limited^11^.

These studies underscore the complex relationship between OT and social behavior and may offer a potential underpinning for connecting increases in OT release during the *randori* component in martial arts, as this high-intensity, free-style dyadic interaction, involving a simultaneous attack at and defense against one’s opponent, clearly requires very high levels of social visual information processing and social interaction. Traditional martial arts practice involves an intensive dyadic effort during a round of sparring, while capitalizing on interpersonal synchrony and social group cohesion. This combination of pro-social synchronous behaviors with competitive sparring may both contribute to increased OT responsivity during a martial arts session.

The significantly greater post-grappling OT response, as compared to sparring, may also be informative. A recent study investigated OT release and associated neural responses during hand- or machine-administered massage in 40 adult male subjects^44^. The authors reported that hand-administered massage increased plasma OT release and activity in brain regions involved in social cognition and reward. Additionally, whereas previous research has demonstrated no difference between lone running and lone sexual stimulation in terms of salivary OT increase^20^, the present findings suggest that the intensive interpersonal contact may be especially conducive to OT response. Grappling *randori* naturally involves a continuous, intensive tactile interaction between the trainees, and, despite the competitive nature of the interaction, requires a significant degree of prosocial cooperation and support throughout the training process. Furthermore, the greater OT response in grappling as opposed to the sparring group, irrespective of expertise, suggests that this response is likely not related to greater anxiety or stress, which would be expected to be greater in the novice group (although this assumption should be directly evaluated in future research).

Mutually agreeable touch, in general, as opposed to non-touch relaxation methods, has been demonstrated to reduce anxiety and impart beneficial effects for social development^45^, with interventions such as massage therapy reducing anxiety in the elderly^46^ and aggression in adolescents^47^. Additionally, it has been shown that greater parental touch is predictive of a greater rise in offspring’s salivary OT following a dyadic interaction^48^, and preliminary research has indicated that lower OT levels may be predictive of the level of touch an individual seeks (e.g., the extent to which dog owners pet and touch their canine companions)^49^. Thus, given that both overt relaxation-intended touch (e.g., massage therapy) and non-focused touch (e.g., dog petting and interpersonal tactile interaction) lead to increased OT, it is not surprising that the extensive tactile interaction involved in grappling may lead to enhanced OT response.

Several limitations of the present study, as well as potential venues for future research, should be acknowledged. First, as suggested above, there could be several explanations for the increase in OT levels during training, such as an exercise-induced OT response and/or a more complex mediation by increasing the salience of social information. Future studies may be able to disentangle these potential mechanisms by comparing, for example, aerobic activities that require attention to socially-relevant information for optimal performance with those that do not require social interactions. Second, due to the “rough” nature of the dyadic interaction during *randori*, it was not possible to monitor physiological parameters of physical exercises. However, as indicated above, simulations of non-contact high-intensity *randori* indicated that participants in both groups maintained heart rate zones comparable with high-intensity interval training. Therefore, the significantly higher OT response during grappling, as compared to sparring, cannot be explained simply by differences in training intensity, but rather likely occurred due to the larger tactile stimulation occurring during the former type of training. Nonetheless, it would be informative to adapt physiological measure of exercise intensity levels to be used during martial arts training, in order to examine potential relationships of these parameters with increases in OT.

Additionally, the current study did not include any emotional or behavioral measures that may be related to OT response. Indeed, a recent study employed exercise-induced OT stimulation in patients with craniopharyngioma (epithelial tumors thought to impair OT production and release), to examine whether altered oxytocin levels would account for affective and emotional dysfunction^50^. The authors reported a positive association between baseline OT and trait anxiety and a negative association between OT response to exercise and state anxiety. This study underscores the complexity of the association among exercise, OT, and emotional functioning and paves the way for future studies, requiring multilevel assessments (e.g., physiological, behavioral, and functional) in order to more fully understand this complex relationship.

It should also be noted that the use of saliva to assess OT has not been without criticism since its early application in neuroendocrinological research. For example, some researchers suggested non-specific binding to non-OT antibodies^51^ and others raised the issue of questionable correlation with plasma OT^52^. In recent years, however, there have been substantial improvements in OT testing kits^38,53^, reducing potential non-specific binding. Research has furthermore demonstrated associations between salivary OT and brain activation in areas rich in OT receptors^54^, as well as increases in salivary OT following intranasal administration^55^. Importantly, consistent with the current study, recent studies have demonstrated salivary OT response to physical activities in primates^56^ and humans^50^. Thus, despite reasonable early criticisms, salivary OT has become in recent years a well-researched and accepted measure across numerous labs around the world.

Finally, the present cross-sectional study was designed to examine the immediate OT response to training, rather than tracking changes in OT release over time. Although the inclusion of beginner and advanced trainees bear some relevance to this issue, the large individual differences inherent in OT production may have prevented meaningful comparisons. Thus, prospective studies following martial arts trainees over time are needed to examine whether acquisition of expertise in this field may lead to differential OT responses.

In sum, the present study is the first to examine the effects of martial arts training on OT response. Findings demonstrated that both types of training, but especially the component involving intense tactile interaction, resulted in significant release of salivary OT. Traditional martial arts typically involve a dyadic, stimulating, and prosocial physical activity and have gained increasing popularity in the last several decades, due to demonstrated positive effects on cognitive functions, self-regulation, and sense of well-being^26,27^. Given the underlying philosophy of traditional martial arts, emphasizing during training the importance of benevolence, humanity, and self-restraint^28^, it is not surprising that OT release is a potential mechanism underlying its beneficial effects. This stimulating physical activity can be readily integrated into diverse community and clinical settings, and, with additional research and adaptation to clinical populations, may potentially offer therapeutic applications in disorders of social dysfunction.

## Methods

### Participants

Participants were recruited from several martial arts schools across the central region of Israel. A total of 68 healthy males (mean age = 27.1, SD = 12.6) participated in the study, of which 30 were advanced martial artists (black belts) and the rest beginners (white or yellow belts). Forty participants practiced DSJJ (21 black belts) and 28 practiced BJJ (9 black belts). Participants were excluded if they had an identifiable medical or psychiatric condition or taking current or regular medications. They were asked to refrain from alcohol, caffeine, or nicotine consumption on the day of the experiment. All methods were performed in accordance with the relevant guidelines and regulations. All participants gave written informed consent after receiving a full explanation of the research according to procedures approved by the Institutional Review Board at Bar-Ilan University, the Israel Ministry of Education Ethics committee, and the Helsinki Ethics committee of Hadassah Hospital in Jerusalem, as required by the Ministry of Health. For participants under 18 years of age, written informed consent was also obtained from their parents.

### Saliva sampling and oxytocin measurement

Salivary OT measures have been validated across various studies and become a common practice in recent years. For example, salivary OT measurements have been shown to correlate with plasma OT^57^ and have demonstrated consistency across the life-course^58^. Additionally, increasing OT artificially was shown to parallel increases in salivary OT^55^. Thus, given that the present study focused on repeated measures of OT over a short duration, salivary OT was deemed the most efficient and effective method of assessment.

Three saliva samples were collected at baseline, immediately after the peak training intensity, and following a cool-down period. Participants gave saliva by passive drooling into a clean 5 ml tube. If the participant had difficulty producing sufficient saliva, they were instructed to massage their jaw and imagine food on their tongue. Participants were told that they may drink water immediately following saliva production but refrain from drinking until following the next saliva sampling. All samples were then stored at −20 °C.

In order to precipitate the mucus, samples underwent three freeze-thaw cycles: freeze at −80 °C and thaw at 4 °C. After the fourth cycle the tubes were centrifuged twice at 1500 g (4000 rpm) for 30 minutes each. Supernatant was collected and the aliquots stored at −20 °C until assayed. In order to increase the sensitivity, the liquid samples were concentrated four times by freeze-drying for 3–4 days to yield a powder. Prior the freeze-drying procedure the samples were stored at −80 °C, for at least three days. OT concentration was measured using a commercial OT Enzyme-Linked Immunosorbent Assay (ELISA) kit (Cayman Chemicals, Ann Arbor, Michigan, USA). The kit provides quantitative *in vitro* assay for OT in human saliva. The dry samples were reconstructed with the assay buffer immediately before the assay. Measurement was done in duplicate, according to the kit’s instructions. The concentration of OT in the samples were calculated using MatLab (The Natworks, Natick, MA) according to relevant standard curves. The intra-assay coefficients of samples and controls were less than 12.4% and 14.5%, respectively.

### Procedure

**Figure 3:**
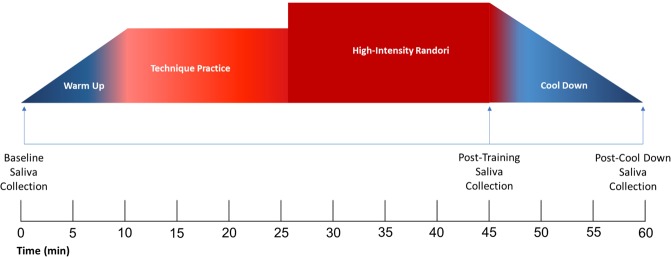
Schematic representation of the martial arts session and salivary sampling for oxytocin.

To maximize attendance, data collection was scheduled for a specific date, and participants were provided with a summary of the research by their instructors during the previous week. All data collection took place during the summer months in the evening after dark, to control for seasonal/daily hormonal fluctuations. After the researchers explained the study procedures and obtained written informed consent (minors provided parental informed consent prior to the scheduled date), demographic information (i.e., age, fitness to participate, martial arts trainee level) was collected. Participants then provided a baseline saliva sample and began the martial arts training session. Through prior coordination between the researchers and martial arts instructors, the sessions were standardized across schools to include the following components: an approximate 10-minute warm-up, 15-minute technique practice, 20-minutes of high-intensity *randori*, and a 15-minute cool-down period (see Fig. 3). Thus, the training sessions were generally similar, except for the *randori* component, such that DSJJ trainees engaged in a typical “punch-kick” sparring, whereas BJJ engaged in ground grappling. Given the rough interactions during the *randori* component, it was not possible to monitor physiological parameters of physical exercises using standard heart rate (HR) monitors, such as arm bands or chest straps. However, simulation of non-contact high-intensity *randori* indicated that participants in both groups maintained 1–3 min periods at 85%-95% of individual HR_max_, alternating with 30–60 sec periods at 60%-70% of individual HR_max_ (estimated using standard HR zone calculation). This form of physical exercise would be considered high-intensity interval training (HIIT), interspersed with active recovery periods^59,60^. The second saliva sample was obtained immediately following the high-intensity *randori*, and the third sample was taken following the cool-down period. At the end of the session, researchers provided additional information about the nature of the study and answered any questions from the participants.

### Data analysis

To test for differences in OT response, we fitted the data with a Linear Mixed Model (LMM), using R’s lme4 package^61^. The advantage of mixed effects models is that they account for variability between subjects and correlations within the data and are superior to Repeated Measures ANOVA in handling missing data^62^. The fixed factors of the fitted models included type of *randori* (sparring vs. grappling), trainee level (beginner vs. advanced), and time of saliva collection (baseline, peak-training, cool-down), as well as their interactions. A random intercept for subject was also included in the model. Significant results were followed by post-hoc analyses with Bonferroni correction. We also employed standard formulas^63^ to compute the areas under the curve with respect to ground (AUC_G_) and with respect to increase (AUC_I_) and used *t*-tests to compare the total OT production between the groups. Because several participants did not produce sufficient saliva for analysis at different time points (four participants at baseline, seven following peak training, and twelve following cool-down), missing data was handled by repeating the analyses using maximum-likelihood expectation-maximization^64^, which yielded virtually identical results.

## Data Availability

Data are available upon request.
